# Enamel Pearls Implications on Periodontal Disease

**DOI:** 10.1155/2015/236462

**Published:** 2015-09-06

**Authors:** Elton Gonçalves Zenóbio, Thaís Ribeiral Vieira, Roberta Paula Colen Bustamante, Hayder Egg Gomes, Jamil Awad Shibli, Rodrigo Villamarin Soares

**Affiliations:** ^1^Department of Dentistry, PUC Minas Master Program of Implantology and Periodontology, 500 Dom José Gaspar Avenida, 46 Hall, 30535-610 Belo Horizonte, MG, Brazil; ^2^Department of Dentistry, PUC Minas Master Program of Periodontology, Belo Horizonte, MG, Brazil; ^3^Department of Dentistry, PUC Minas Master Program of Implantology, Belo Horizonte, MG, Brazil; ^4^Department of Dentistry, PUC Minas, Belo Horizonte, MG, Brazil; ^5^Dental Research Division, Department of Implantology and Periodontology, School of Dentistry, UNG, Guarulhos, SP, Brazil

## Abstract

Dental anatomy is quite complex and diverse factors must be taken into account in its analysis. Teeth with anatomical variations present an increase in the rate of severity periodontal tissue destruction and therefore a higher risk of developing periodontal disease. In this context, this paper reviews the literature regarding enamel pearls and their implications in the development of severe localized periodontal disease as well as in the prognosis of periodontal therapy. Radiographic examination of a patient complaining of pain in the right side of the mandible revealed the presence of a radiopaque structure around the cervical region of lower right first premolar. Periodontal examination revealed extensive bone loss since probing depths ranged from 7.0 mm to 9.0 mm and additionally intense bleeding and suppuration. Surgical exploration detected the presence of an enamel pearl, which was removed. Assessment of the remaining supporting tissues led to the extraction of tooth 44. Local factors such as enamel pearls can lead to inadequate removal of the subgingival biofilm, thus favoring the establishment and progression of periodontal diseases.

## 1. Introduction

Inflammatory periodontal diseases present an etiological cause which is primarily determined by a bacterial biofilm and its subproducts. Multiple factors influence the quality of this biofilm as well as its pathogenic potential. The basis of periodontal therapy can most commonly be found in the maintenance of a specific microbiota through the control of the dental biofilm and decontamination of the root surface [1, 2].

Root anatomy has been reported in the literature as a highly relevant predisposing factor in the installation and perpetuation of periodontal diseases. In this context, a number of authors have given priority to in-depth knowledge of root morphology [3–6]. In particular, the presence of enamel pearls can affect mechanical control of dental biofilm, since they serve as niches which retain bacterial biofilm and dental calculus [7].

Ectopic globules of enamel, or the so-called enamel pearls (EP), can be either internal or external, with the former being more common and usually found at a cervical or coronary location on the root surface. These globules are most frequently located near the enamel-cementum junction. EP origin is probably related to a localized developmental activity of Hertwig's epithelial root sheath (HERS) cells that remained adherent to the root surface as the root development proceeds. HERS cells differentiate into functioning ameloblasts which deposit an enamel organic matrix on the root surface [7–9]. Additionally, a recent report discussing the presence of multiple EP in two siblings raised the possibility of a hereditary association in the formation of EP [10].

These deposits predominantly consist of enamel and rarely present pulp tissue in their composition. They are most frequently located in bifurcations or trifurcations of multiradicular teeth but can also appear in uniradicular teeth. The maxillary molars are affected more frequently than mandibular molars [8, 11]. Nearly three-fourths of the EP might be found in the maxillary third molars, although their appearance in mandibular third and second molars may also be observed [6]. These circular formations can achieve 0.3 to 2.0 mm in diameter, in the majority of the cases only one EP incidence can be detected in each tooth, and two or more pearls located on the surface of a single tooth are rarely observed [6, 9, 12].

The prevalence of EP may differ depending on the method used to detect them. In a clinical exam performed by Kerr [13], EP were found in at least 1% of all extracted teeth and in 2% of the maxillary molars. In another report histological exam of extracted teeth revealed that nearly 15% of all teeth and 54% of maxillary molars presented EP [7]. Moskow and Canut [6] demonstrated that these globules can be diagnosed by radiographic examination. Their study reported a mean incidence of 2.6% and a variation of 1.1% to 9.7% depending on tooth type and location.

There is evidence suggesting that the clinical significance of EP may be related to periodontal disease. These nodules contribute to local deepening of periodontal pockets because, in their presence, the attachment of the periodontal ligament does not occur properly [14]. The periodontal pocket, when present, extends apically to the EP generating difficulty on biofilm control and restricting access for scaling and root planning. In this context, the presence of EP in various periodontal lesions has been previously described [6]. EP must be removed or undergo intervention to allow access to the area for proper biofilm control by patients and professionals in order to prevent future attachment loss [5].

## 2. Case Description and Results

**Figure 1:**
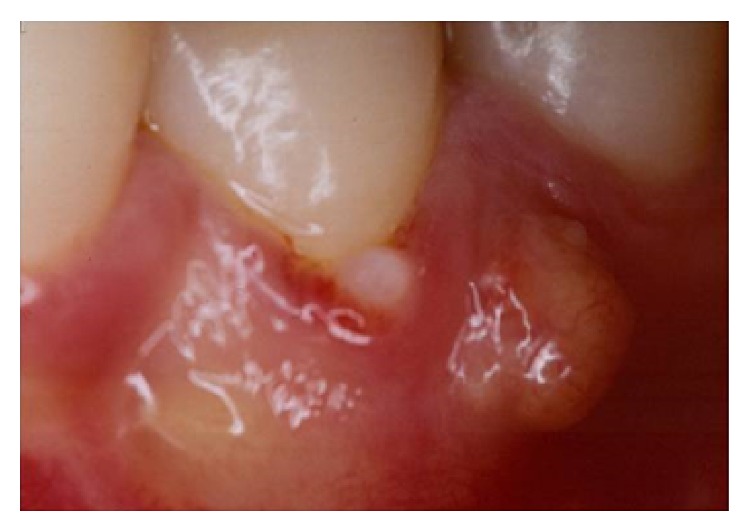
Gingival abscess and suppuration in the premolar area.

**Figure 2:**
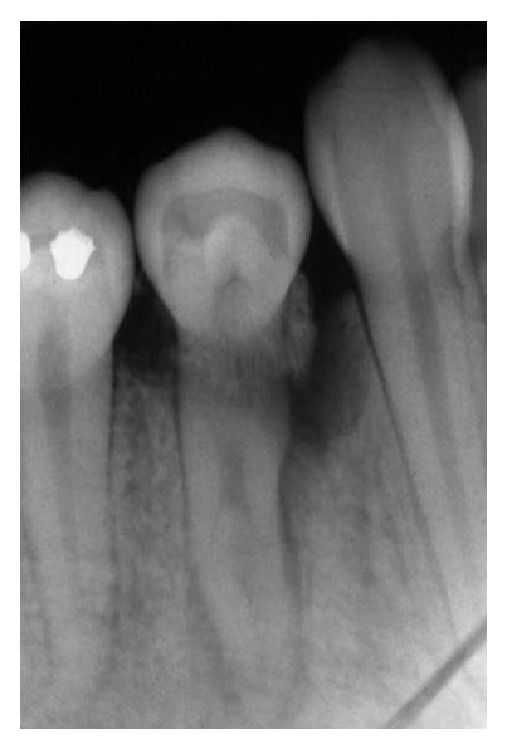
Radiograph of tooth 44. The presence of a radiopaque structure can be observed around the cervical region.

**Figure 3:**
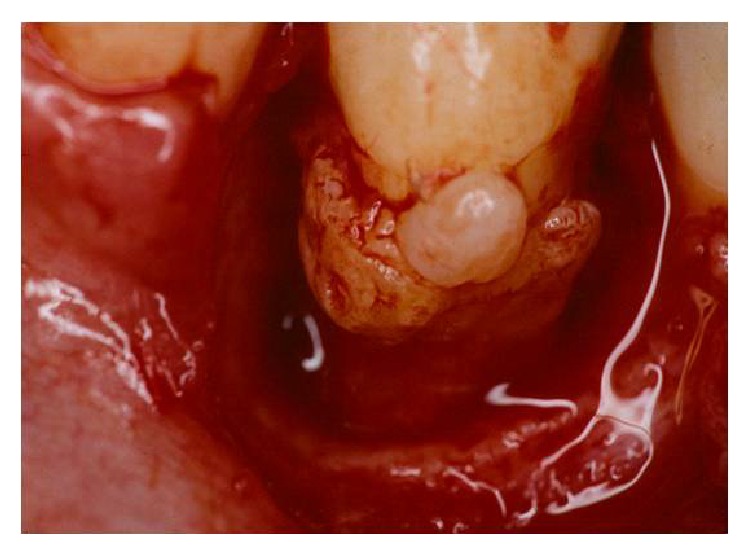
Surgical exploration. The presence of EP associated with extensive bone loss can be observed.

**Figure 4:**
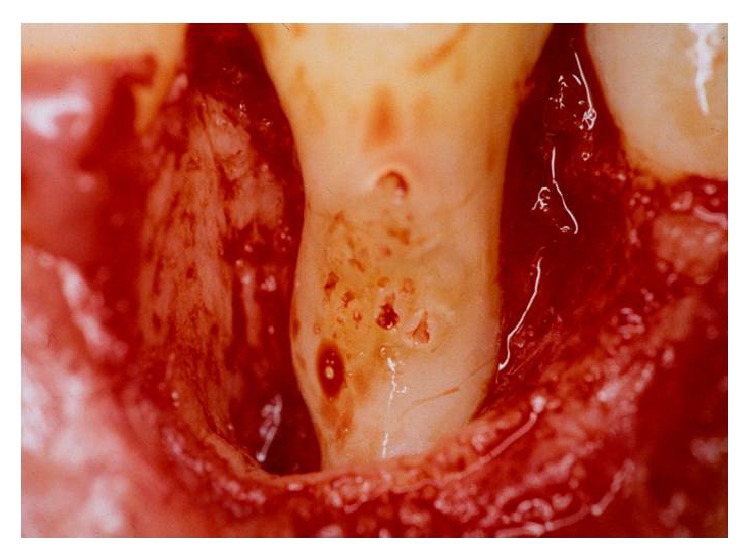
Assessment of the remaining supporting tissues.

A 18-year-old female patient complaining of pain in the right mandibular region was examined at Department of Dentistry, PUC Minas. During the clinical examination, pulp vitality and absence of mobility were shown, probing depths of 8.0 mm at the mesiobuccal, 7.0 mm at the distobuccal, and 9.0 mm at the midbuccal sites were encountered, and, additionally, intense bleeding, suppuration, and the presence of a gingival abscess could also be observed (Figure 1). Radiographic examination of lower right first premolar revealed the presence of a radiopaque structure around its cervical region (Figure 2). Surgical exploration was performed by means of a conventional gingivoperiosteal flap and the presence of EP associated with extensive bone loss was observed (Figure 3). After removal of anatomical alterations and assessment of the remaining supporting tissues (Figure 4), the extraction of tooth 44 was indicated and conducted.

## 3. Discussion

Mechanical factors which favor the retention and growth of the dental biofilm act as secondary etiological factors of periodontal diseases [15]. They are usually derived from abnormal anatomical characteristics of the roots and/or restorative interventions (i.e., inadequate or excessive margins of subgingival restorations) that might increase bacterial adhesion and growth on dental surfaces and on pocket epithelium [3] leading to periodontal diseases [4].

The presence of EP associated with a number of periodontal lesions has been reported [5, 7, 16] and although small EP can be inadvertently removed during scaling and root planning, larger ones may be an obstacle to appropriate root instrumentation and additionally and clinically and at radiographic exam incorrectly diagnosed as dental calculus [6].

There is scientific consensus that EP favor the installation or at least an increase in the severity of isolated periodontal problems [5–7, 11, 14]. Therefore, this anatomical anomaly demands a more careful approach and a precise diagnosis since unidentified EP can possibly initiate or aggravate periodontal diseases leading to unfavorable tooth prognosis.

## 4. Conclusions

Many failures in periodontal treatments are related to inadequate evaluation of the teeth with distinct anatomical characteristics and incorrect diagnosis. Therefore, a detailed assessment of variations on teeth anatomy is important to obtain a correct diagnosis, more predictable and reliable prognosis to select the appropriate periodontal therapy.
